# Quality of life post oral rehabilitation with complete short arch among South Indian patients

**DOI:** 10.6026/97320630018531

**Published:** 2022-06-30

**Authors:** Vatika Agarwal, Velayudhan Ashok, Subhabrata Maiti

**Affiliations:** 1 Saveetha Dental College and Hospitals, Saveetha Institute of Medical and Technical Sciences, Saveetha University, Chennai 600077, Tamil Nadu, India

**Keywords:** Oral rehabilitation, tooth loss, tooth wear, short dental arch, complete dental arch, quality of life

## Abstract

Oral rehabilitation is essential in patients having multiple missing teeth, to restore esthetics and function. However replacement of complete dentition may not be possible in high risk patients or patients of low income group. The concept of SDA can be utilized in such patients to increase affordability and avoid over restoring. The aim of this study was to understand the prevalence of the requirement of oral rehabilitation according to age and gender of the individual as well as to compare the quality of life of patients rehabilitated with complete and short dental arches. Case sheets of around 28,000 patients were reviewed from March 2019 to June 2020 out of which 113 patients were undergoing oral rehabilitation. To eliminate bias all patients affected by the disease were included in the study. Epidemiological data of the patient along with their ongoing treatment was collected and tabulated in MS Excel sheet. Amongst the patients 36 patients were selected using simple stratified sampling and patients were asked to report their quality of life on a VAS scale using a quality of life questionnaire. The data was then analyzed using IBM SPSS software version 23. The prevalence of oral rehabilitation in males was 53.98% while in females it was 48.02%. Age group of 41-60 years was found to be most commonly undergoing oral rehabilitation. The most common extent of rehabilitation was 2nd molar - 2nd molar, around 23% patients underwent rehabilitation from 1st molar-1st molar and around 10% patients underwent rehabilitation from 2nd premolar-2nd premolar. Patient satisfaction was greatest in patients with complete arch restoration when compared to short dental arch restoration. Amongst SDA restoration patients rehabilitated till the first molar had greater satisfaction than patients rehabilitated till second premolar. With increase in lifespan the need for oral rehabilitation has increased, however it is not always possible or advised to restore the complete dentition of an individual. The present study helps us understand the prevalence of oral rehabilitation according to the gender and age of the patients as well as use of newer concepts such as SDA in the management of high risk patients. Within the limitations of the present study patient satisfaction was greater in complete arch restoration compared to SDA. Thus it is important to understand the requirements of the patient and rehabilitate accordingly, so as to provide the patient with the best esthetics and function possible.

## Background:

Every individual develops 14 to 16 functional units, with the exception of patients having developmental disorders like ectodermal dysplasia, hypodontia etc. However, despite patient management in the form of preventive and restorative dental care, problems can accumulate, leading to carious or periodontally involved teeth which may lead to tooth loss. A fundamentally occurring complex issue in dental restorations is determining the number of teeth that should be saved or replaced in order to achieve desirable oral function. The traditional restorative approach emphasised molar support as a means of preventing temporomandibular joint problems and occlusal instability. Overtreatment occurs as a result of the obligation to preserve or substitute every missing tooth [1-2]. Hence the concept of shortened dental arch was introduced. The term 'shortened dental arches'(SDA) was initially described in 1981 by Arnd Kayser, a Dutch prosthodontist [3][4]. It refers to a specific type of dentition that has an intact anterior region but fewer occluding pairs in the posterior region [5][6][7]. This is a common occurrence because molar teeth are at a high risk of decay and are frequently affected by both caries and periodontal diseases [8] [9], while premolars and anterior teeth tend to survive longer [10][11] [12][13]. Traditionalists believe that the goal of restorative dentistry is to preserve the complete dental arch, however the concept of SDA is used in the management of elderly and middle aged patients with reduced dentition. This was based on the current concept of healthy and physiologic occlusion proposed by Ash & Ramfjord (1995) [14][15] which stated that A healthy occlusion needed to:

1. Be absent of pathologic manifestations

2. Have satisfactory aesthetics and functions like chewing

3. Have variety in form and function

4. Have the ability to adapt to changing circumstances

Variation in the function and form allowed the number of teeth to be different from the traditional number, 28, according to the patient's requirements. This along with the concept of adaptability justified the acceptance of concepts like SDA in the restoration of the oral cavity of selected patients depending on the functional and esthetic requirement of the individual. As limited treatment goals can also adequately satisfy a patient's expectations [16] ,SDA should be considered as a viable treatment option especially in patients who have an increased predisposition for caries and periodontal disease. This is especially true in the elderly, who often have plenty of dental issues in addition to other age-related risks. Management such patients using complete dental arches may technically be possible but not advisable considering the cost-time-benefit analyses. Therefore, it is of interest to understand the prevalence of the requirement of oral rehabilitation according to age and gender of the individual as well as to compare the quality of life of patients rehabilitated with complete and short dental arches.

## Materials and methods:

This study was carried out in a university setting at Saveetha Dental College and hospital, Chennai, India by the department of Prosthodontics and Implantology. The study consisted of a data collector and 1 data reviewer. The disadvantage of the study was its geographical limitation and high dropout rate. This study was approved by the institute ethical board committee. Data of patients visiting the department of Prosthodontics and Implantology at Saveetha Dental College and Hospital from June 2019 - March 2020 was collected by method of simple random sampling. A total of 28,000 case sheets were reviewed out of which 113 patients had undergone full mouth rehabilitation. Epidemiological data of the patient along with their ongoing treatment was collected and tabulated in MS Excel sheet. Amongst the patients 36 patients were selected using simple stratified sampling and patients were asked to report their quality of life based on the Oral Health Related Quality of Life questionnaire. The oral health related quality of life (OHRQoL) can be measured with the Oral Health Impact Profile (OHIP)[17]. It consists of 19 criteria's which are divided into 7 domains (functional limitation, physical pain, physiological discomfort, physical disability, physiological disability, social disability and handicap). It is a complex scale hence we chose to use the OHIP-14 scale which is a smaller subset. Additionally it has also been validated in the Indian population [18][19]. A score consisting of "very often (4)", "fairly often (3)", "occasionally (2)", "hardly ever (1)", and "never (0).was given for the criteria's and the data analysis was done. External validity is that it is applicable to the South Indian population. The data collected was entered in MS Excel spreadsheet and tabulated. The data was imported in SPSS software version 23 and variables were defined. Statistical analysis of data was carried out using descriptive frequency analysis, chi square test, one way ANOVA and the Tukey HSD post hoc test. Independent variable was tooth wear factors like attrition, abrasion, para functional habits and dependent variable was age, sex, type of procedure. The OHIP scores given by the patients for the oral rehabilitation were tabulated and the mean, standard deviation and P value were calculated for each criteria using SPSS version 23 software.

## Result and Discussion:

A total of 28,000 case sheets were reviewed amongst which 113 patients were undergoing full mouth rehabilitation at the department of prosthodontics and implantology. In the present study it is observed that male was undergoing oral rehabilitation more frequently than females. Amongst the 113 patients undergoing oral rehabilitation, 53.98% (61) were male and 46% (52) were female (Figure 1 - see PDF). It was also seen that patients in the age group 41-60 years (54%), were undergoing oral rehabilitation more frequently than patients of other age groups while patients below 20 years of age were undergoing oral rehabilitation the least (0.9%) (Figure 2 - see PDF). In the present study it is also observed that most oral rehabilitations were extending from the 2nd molar to 2nd molar (66.37%), (23.01%) were extending from the first molar-first molar, (10.62%) from 2nd premolar to 2nd premolar (Figure 3 - see PDF). The association between age of the patient and the extent of the oral rehabilitation is statistically not significant (Pearson Chi square value- 12.241; P value- 0.057). However, it is observed that patients of age 41-60 years popularly were rehabilitated from 2nd molar - 2nd molar (Figure 4 - see PDF). The scores reveal that the mean value for all the criteria's in the OHIP scoring chart are on a lower side for the complete arch restoration compared to the restorations extending to 1st molar compared to restorations extending to 2nd premolar. Also the P value was obtained by statistical analysis using SPSS version 23. The P-value was 0.001 and hence the data was statistically significant. (Table 1 to 3 - see PDF).

Multiple tooth loss or wear usually necessitates oral rehabilitation. Teeth loss can be caused by dental caries, periodontal diseases, traumatic injuries, and so on, whereas tooth wears are caused by attrition, abrasion etc. A study conducted by [20][21] [22] concluded that periodontal disease was found to be the leading cause of tooth loss in people, making it one of the most common reasons for oral rehabilitation.. In the present study it is observed that men were undergoing oral rehabilitation more frequently than women. This may be due to greater prevalence of periodontal disease in males compared to females [21,23] [24]. Increased prevalence of periodontal diseases in men may be due to increased frequency of deleterious habits like smoking and alcohol consumption. Similar results were also found in a study conducted by [25][26]. Males usually have poorer oral hygiene compared to females which may also be a cause for increased tooth loss and hence greater need for replacement may be present in males [27][28]. Other causes of tooth loss like traumatic injuries are also more frequently found in men. This may be due to greater participation in activities like driving or aggressive sports [27,29] [30]. Tooth wear which is a common cause of need for oral rehabilitation is also found more frequently in men. This could be due to the increased masseter function, mass of muscle fibre, and strength of ligament [31][32]. In the present study it was also observed that the age group undergoing oral rehabilitation largely comprised patients of age 41-60 years. This may be due to the increased incidence of multiple tooth loss due to increased incidence of periodontal disease at this age [33-34] along with other factors like functional tooth wear. According to a study by [31][35] tooth wear increases with age, hence a greater number of patients within the age group 41-60 years may require oral rehabilitation. According to [31, 36][37] tooth wear increases with age due to prolonged duration of use due to increased retention of teeth in individuals. However patients over 60 years of age didn't frequently undergo oral rehabilitation probably due to decreased financial resources and reduced ability to sit for long durations of dental treatments. Patients under 20 years of age may occasionally require oral rehabilitation due to genetic disorders like ectodermal dysplasia or occasionally due to trauma. With time there has been an increase in oral hygiene awareness amongst the youth, hence very few patients below the age of 20 may actually require oral rehabilitation due to loss of teeth because of dental caries or periodontal diseases.

In the present study, it was observed that most patients underwent oral rehabilitation from 2nd molar - 2nd molar; this may be due to the fact that molar teeth are at high risk and are frequently lost due to caries or periodontal disease. A study conducted by [31, 36, 38][39] found that posterior teeth had a higher plaque index and showed greater inflammation when compared to anterior teeth, hence they were at a greater risk of dental caries or periodontal disease. Several other researchers also concluded that posterior teeth were more prone to caries due to their anatomical form containing multiple fissures and grooves [40] or occasionally due to the presence of impacted third molars [41] causing increased incidence of decay at their proximal surface. The traditional need and mind set of restoring the complete dentition also played a role in the fact that most patients received 2nd molar-2nd molar restorations. Occasionally some patients were restored from 1st molar- 1st molar or 2nd premolar - 2nd premolar. This is due to the fact that most anterior teeth with a few occluding posterior teeth often fulfill the esthetic and functional requirements of the patients. According to a study by (3, 41), patients have enough adaptive capacity to maintain required oral function in shortened dental arches if at least four occluding units are present. In the present study a quality of life assessment was done using a telephonic survey. It was observed that patients who received complete arch restoration were more satisfied than patients receiving shortened dental arch restoration. Similar results were found in a study conducted by [42] who concluded that 'those oral function problems increased with decreasing functional units'. Many clinicians were sceptical of the SDA concept because they believed that losing molars was linked to poor masticatory function and could lead to mandibular displacement [43- 44]. SDA has also been linked to an increased risk of temporomandibular joint changes [45- 46]. Within shortened dental arch restorations patients receiving restoration upto first molar were more satisfied in comparison to patients receiving restoration up to 2nd premolar. Similar results were also found in a study conducted by [47] on the Tanzanian population concluded that patients when restored with at least 1 pair of occluding molars had lesser complaints (3%) when compared to patients having only occluding premolars(98%). However based on 6-year follow-up studies of subjects with a SDA condition by [48-49] it was concluded that;

[1] SDA is capable of providing adequate occlusal stability.

[2] SDA patients don‘t display signs and symptoms of mandibular dysfunction

[3] In terms of chewing ability and dental appearance, patients with SDA have adequate oral comfort.

[4] In SDA, a free-end RPD improves oral function.

Similarly a study conducted by [50] inferred that the SDA although widely accepted isn't widely practiced in the United Kingdom. Around 82% SDAT patients displayed good oral function, comfort, and well-being. SDA did not cause overloading of the TMJ or the teeth, according to a study conducted by [51] which concluded that the neuromuscular regulatory systems are effective in regulating the maximum clenching force in various occlusal situations [52]. Although in this study it is revealed that patients were more satisfied with complete arch restorations and the data is statistically significant, the data may not be clinically significant as this was only a questionnaire based study purely based on the patient's perception. No tests were carried out testing the functional efficiency of the patients. Though functionally ok most patients may be subconsciously dissatisfied due to the presence of less :new: teeth in the oral cavity. Other limitations of this study were that it was geographically limited. The sample size was also less. Hence studies with larger sample size, longer duration of follow up and functional tests are required to achieve conclusive data. Despite the fact that the majority of dentists agree with the SDA concept, it is not widely used. The SDA should be included in the treatment planning process of at least high-risk and unaffordable patients, and their needs and demands should be assessed individually. In most patients, restoring the entire arch is necessary for a good aesthetic and functional outcome, but newer research suggests that SDA can meet long-term oral functional demands such as aesthetics, the ability to chew, occlusal and mandibular stability. The SDA concept is compatible with modern dentistry's problem-solving approach and does not contradict current occlusion theories. Hence SDA concept offers a realistic treatment strategy, reducing complex restorative treatments and can be used as a treatment modality in patients with increased risk. This study helps us understand the requirement of oral rehabilitation in the population according to age and gender, so that we can tackle problems like periodontitis and dental caries in those populations at a grass root level. This study also helps us understand the popularity of recent concepts like SDA, which can be considered as a treatment option in the management of selective patients.

## Conclusion:

Within the limitations of the present study it can be concluded that males required oral rehabilitation more frequently than females. It can also be concluded that the most popular age group to undergo oral rehabilitation was 41-60 years. The patient satisfaction level and quality of life, although better in complete arch restorations, was good in patients with SDA too. Hence in high risk patients, unaffordable patients or patients who require distal extension or RPD to restore posterior teeth, it is advisable to give a Short dental arch instead. As a result, it's critical to understand the patients' needs and rehabilitate them appropriately in order to provide the best possible aesthetics and function.
